# Validity of the school setting interview for students with special educational needs in regular high school – a Rasch analysis

**DOI:** 10.1186/s12955-017-0830-6

**Published:** 2018-01-12

**Authors:** Moa Yngve, Michaela Munkholm, Helene Lidström, Helena Hemmingsson, Elin Ekbladh

**Affiliations:** Department of Social and Welfare Studies, Linköping University, Norrköping, Sweden

**Keywords:** Psychometrics, Neuropsychiatric disorder, Dyslexia, Assessment, Instrument development, Person-environment fit, Participation, Support in school, Occupational therapy

## Abstract

**Background:**

Participation in education is a vital component of adolescents’ everyday life and a determinant of health and future opportunities in adult life. The School Setting Interview (SSI) is an instrument which assesses student-environment fit and reflects the potential needs for adjustments to enhance students’ participation in school activities. The aim of the study was to investigate the psychometric properties of the SSI for students with special educational needs in regular high school.

**Methods:**

A sample of 509 students with special educational needs was assessed with the SSI. The polytomous unrestricted Rasch model was used to analyze the psychometric properties of the SSI regarding targeting, model fit, differential item functioning (DIF), response category functioning and unidimensionality.

**Results:**

The SSI generally confirmed fit to assumptions of the Rasch model. Reliability was acceptable (0.73) and the SSI scale was able to separate students into three different levels of student-environment fit. DIF among gender was detected in item “Remember things” and in item “Homework” DIF was detected among students with or without diagnosis. All items had disordered thresholds. The SSI demonstrated unidimensionality and no response dependence was present among items.

**Conclusion:**

The results suggest that the SSI is valid for use among students with special educational needs in order to provide and evaluate environmental adjustments. However, the items with the detected DIF and the SSI rating scale with its disordered thresholds needs to be further scrutinized.

## Background

The concept of special educational needs is internationally used to describe students who temporarily or permanently experience difficulties in their learning. The concept covers students with and without a diagnosis, and includes up to 20% of school-aged youth [1]. Common diagnoses among students with special educational needs are Attention Deficit Hyperactivity Disorder (ADHD), Asperger’s syndrome and dyslexia [2]. Symptoms of these diagnoses often include difficulties to concentrate, follow instructions, organize and conduct tasks, and/or read and write [2–4]. Struggling to complete education successfully may lead to lower self-esteem and poor overall mental health [2, 5], along with higher rates of school-dropout and unemployment due to low academic achievements [2, 6]. Compared with students with physical disorders or without disabilities, students with developmental disorders (ADHD, autism or dyslexia) rate their perceived quality of life significantly lower [7, 8]. Adolescents’ participation in education at school is a vital component of their everyday life and a determinant of health, development and well-being as well as future opportunities in adult life [9]. To achieve full potential for occupational participation in education, students’ opportunities as regards their learning conditions and engagement in school activities are critical [10]. Students should be provided with appropriate adjustments and support [11] in areas such as writing, reading, knowledge gathering, and practical tasks, as well as support in initiating and organizing school activities and reminders to perform tasks at appropriate times [12]. Focusing on the social environment and the learning context, e.g. communication and participation within the classroom, is also important to emphasize [13]. Student’s unique abilities and the characteristics of the specific school environment must be considered and should form the basis for adjustments to give students with different types of disabilities equal opportunities for participation in education [14].

By assessing the student-environment fit, the interaction between the student and the school environment, information reflecting the student’s occupational performance is generated. The School Setting Interview (SSI) [15] is an assessment instrument that assesses student-environment fit and is theoretically based on the Model of Human Occupation [10]. The SSI consists of a student-centered interview and an accompanying rating scale including 16 items of everyday school activities. The assessment takes about 40 min to complete and identifies possibilities, hindrances and potential needs for adjustments concerning students’ participation in school activities [15].

The SSI was initially developed for students with physical disabilities [16] and a psychometric study supported evidence of construct validity. However, the study revealed a need for more challenging items and a refinement of the scoring in the rating scale [17], which thereafter was developed from a three-step to a four-step rating scale in version 2 [18]. Findings and input from professionals with experience of the SSI were used to develop the SSI to make it applicable to students with other difficulties than just physical [19]. Even though the SSI, version 3.1 [15], has been successfully used for students with different difficulties, the construct validity has not yet been evaluated for students with special educational needs. This calls for psychometric evaluation of the internal construct validity of the scale. Depending on the characteristics of the student, different school activities and environmental factors are experienced as more or less challenging. It is hypothesized that students with special educational needs, often involving difficulties with concentration, organization and finishing tasks, will experience more challenges, i.e. low student-environment fit, in school activities such as “Remember things”, “Read”, “Take exams” and “Write”. On the other hand, they may experience high student-environment fit regarding “Access the school” and “Go on field trips”.

The Rasch measurement model is a mathematical approach belonging to modern test theory [20], and has become established as the standard for modern psychometric evaluations of outcome scales [21]. The analysis addresses several measurement issues and aspects in addition to those in classical test theory. It transforms ordinal data to an interval-level variable for detailed investigation of the structure and operation of rating scales [21, 22].

The aim of the present study was to investigate the psychometric properties of the School Setting Interview (SSI) for students with special educational needs in regular high school. More specifically, the aim was to examine whether the SSI items are valid for this group of students, with additional consideration on bias of items by gender and diagnosis, the measurement properties of the SSI rating scale, and whether the SSI measures a unidimensional construct.

## Methods

### Research design

The Rasch measurement model was used to evaluate the psychometric properties of the SSI for students with special educational needs in regular high school. Secondary data, from Swedish governmental projects conducted in 2011-2014 was used. Approval from the Regional Ethics Board in Linköping, Sweden was obtained, study code 2013/409-31.

### Sample and procedure

The secondary data originates from five municipalities, including 12 public high schools with approximately 10.000 students. School staff, in these schools, identified and asked students about involvement in the projects. Potential participants were identified due to inability to reach educational goals and/or noticeable difficulties with planning, problem solving, conducting and/or finishing tasks and/or a high level of school absence. Further, students should be able to speak and understand Swedish. A total of 549 students were included in the projects and gave written informed consent to use their data in research.

**Fig. 1:**
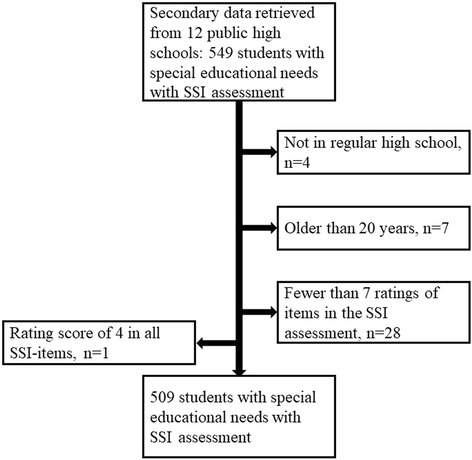
Flowchart of the inclusion of students SSI assessment

Inclusion criteria for participants in the present study were: students in regular high school, ≤ 20 years and at least seven ratings of SSI-items in the SSI assessment. One student obtained the highest rating (rating of 4) in every SSI-item and was excluded since maximum scores do not yield information to the Rasch analysis as the standard errors are infinite and the item responses do not vary [23]. The present sample consists of 509 SSI assessments of students with special educational needs, see Fig. 1.

### Data collection

The secondary data used consisted of a questionnaire with students’ demographic information and assessments of student-environment fit with the School Setting Interview (SSI) [15]. During the SSI assessment, the student was asked to describe his/her functioning in school and potential need of adjustments to meet requirements in different school activities. The SSI items were then rated. Demographic information (age, gender and diagnosis) was obtained through a questionnaire in connection to the interview, see Table 1. The occupational therapists (*n* = 6) and the special education teacher conducting the SSI assessments were trained SSI-assessors.

**Table 1:** Characteristics of the sample (*n* = 509 students)

Age	
Mean, median	17.34, 17
Gender	
Boys *n* (%)	300 (59)
Girls *n* (%)	209 (41)
Diagnosis	
No diagnosis *n* (%)	270 (53)
Neuropsychiatric disorder *n* (%) (e.g. ADHD, Asperger)	112 (22)
Dyslexia/language disorder *n* (%)	100 (20)
Other *n* (%)	27 (5)

### Analysis

The Rasch analysis was performed with the RUMM2030 software [23], using the polytomous unrestricted Rasch model. The objective was to test how well the observed data fit the theoretical expectations of the model, and different fit statistics were examined both statistically and graphically. The Rasch analysis accommodates missing data [21, 22] and SSI assessments with missing ratings of items could be included. The significant level was overall set at *p* < 0.05.

### Targeting and reliability

To evaluate the targeting of the SSI scale, the relationship of persons and items was investigated. For a well targeted scale, the mean sample location should be close to the mean item location (zero) [24, 25]. The reliability of the internal consistency of the scale was evaluated using the person separation index (PSI), analogous to Cronbach’s alpha. The PSI value range from 0 to 1, a value of 1 is the ideal and 0.7 the lowest level of acceptability. The PSI also provides information on how many groups of individuals, strata (statistically distinct groups separated by ≥3 standard errors) the scale can separate between [25–27]. For example, a PSI of 0.2 indicates one strata and a PSI of 0.92 indicates five strata [27].

### Model fit

Fit refers to the extent to which observed responses accord with the mathematical expectations of the model. Summary statistics of invariance of items for this trait were evaluated with chi-square statistics, supporting the required property of invariance when non-significant. Summary statistics also concern item-person interaction presented as the z-score. A perfect fit to the model would have a mean of zero and a standard deviation (SD) of 1, representing a standardized normal distribution [23, 28]. The fit statistics of individuals and items are presented as residuals, considered adequate if they fall in the range of ±2.5 with additional chi-square statistics representing model fit when non-significant. The fit of individual items was also analyzed graphically using an item characteristic curve (ICC). Appraisal of all fit statistics (residuals, chi-square and ICC) determines whether the item is considered to fit or misfit the model [21].

### Differential item functioning (DIF)

DIF was examined to investigate whether the SSI items measured the same ability in the same way across gender (boy/girl) and among students with or without a medical diagnosis within the sample. The presence of DIF was analyzed both statistically, through an analysis of variance (ANOVA), and graphically by the ICC [21–23, 26]. In addition to evaluation of significance, Bonferroni correction was applied, *p* < 0.001. In the presence of DIF, this was adjusted for by splitting the item into two new items [23], one for boys and one for girls, and one for students with a medical diagnosis and one for students without diagnosis, and performing a new analysis of the resolved data set.

### Response category functioning

The category structure (thresholds) of items is considered when investigating polytomous scales. Thresholds, the locations where there is a 50/50 probability of responding in either of two adjacent categories, are consistent with the metric estimate of the underlying construct when presented in an ordered set [21]. In the SSI, the ordering of categories represents an increase of experienced student-environment fit and every item has three thresholds (between categories 1-2, 2-3 and 3-4).

### Unidimensionality and local independence

Unidimensionality of the SSI scale was evaluated by a residual-based principal component analysis (PCA) with a varimax rotation. Eigenvalues between 1.4 to 2.1 for the first component in a PCA have been reported as Rasch-fitting, supporting the assumption of a unidimensional scale [29, 30]. The residual correlation matrix examined response dependency where correlations between items above 0.3 indicate dependence. The Rasch model requires the entire correlation of items to be captured by the latent trait, or it may indicate multidimensionality or response dependence [21, 31].

## Results

### Targeting and reliability

**Fig. 2:**
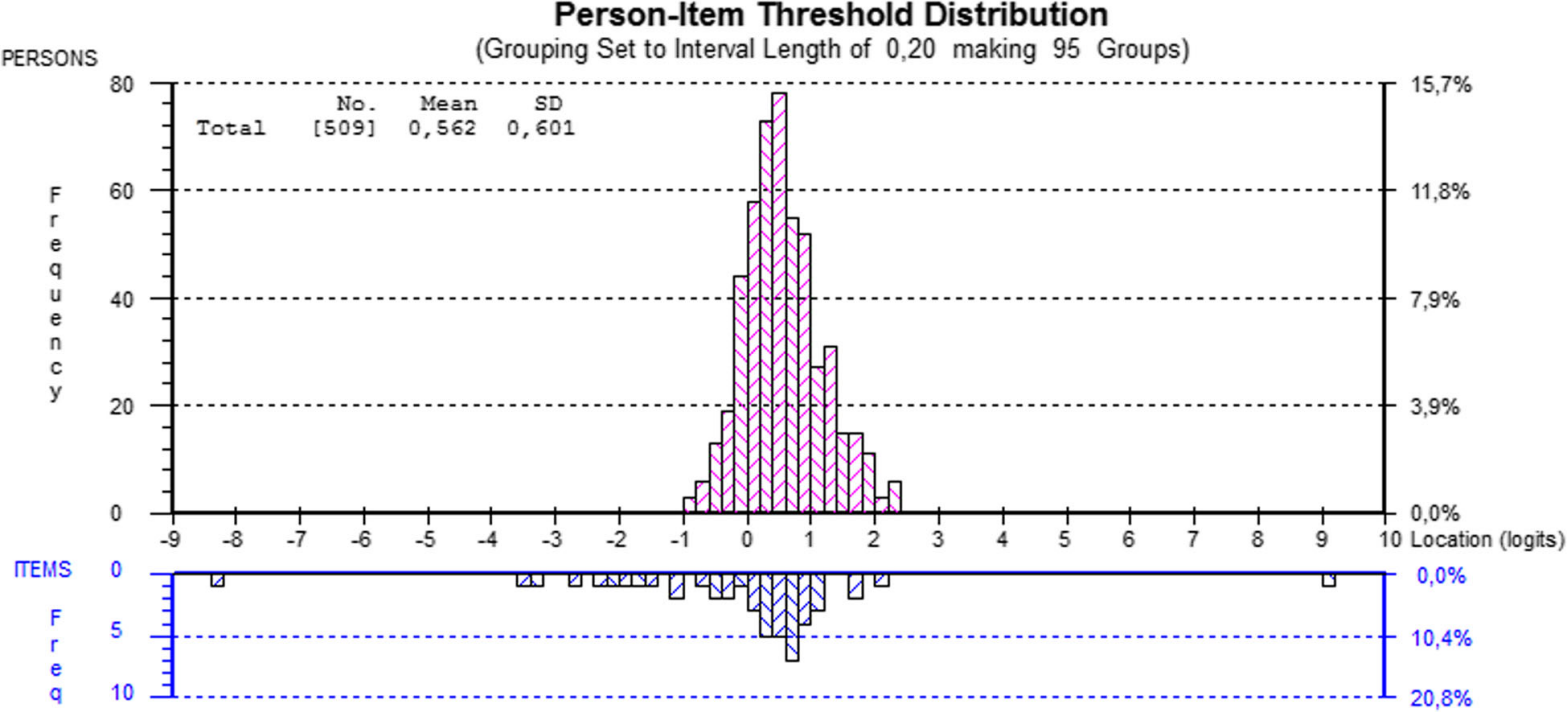
Person-Item Distribution

The SSI items targeted most of the person locations (Fig. 2). Person mean was 0.56 (SD = 0.6), a little to the right of the item mean of 0 (SD = 0.72), indicating that persons exhibit a slightly higher student-environment fit than the difficulty of items represents. The SSI scale worked in an acceptable way (PSI 0.73) and separated students into approximately three groups (2.6 strata).

Illustration of the relationship between student location (*n* = 509, upper panel) and the SSI item response category thresholds (lower panel) on the common latent trait of student-environment fit.

### Model fit

Overall item-fit (mean = −0.15, SD = 1.28) and person-fit (mean = −0.25, SD = 0.73) were acceptable and the non-significant total item chi-square value (*chi sq.* = 135.1; *df* = 112; *p* = 0.07) indicated overall fit to the model. The easiest item was item 13 “Go on field trips” while item 4 “Remember things” was the most difficult. The hierarchical order of item difficulty corresponded well with what were hypothesized to be more and less challenging items for these students. All items, except item 2 “Read” (fit residual of 2.83), had fit residuals within the recommended confidence range of ±2.5, see Table 2. The misfit of item 2 was non-significant. Graphical inspection of the ICC revealed misfit between the observed values and the expected curve of the model in the middle of the trait, indicating poor discrimination. All but two persons displayed good fit and they remained in the analysis.

**Table 2:** Individual Item-Fit of the SSI items (location order) and category response proportions

	Item	Location	SE	Residual	n	Score Category			
						1a) 0	2b) 1	3c) 2	4d) 3
4	Remember things	1.209	0.052	−0.864	509	0.49	0.30	0.09	0.12
1	Write	1.053	0.050	1.081	509	0.44	0.32	0.11	0.13
6	Do homework	0.716	0.045	0.036	472	0.40	0.22	0.12	0.25
2	Read	0.661	0.042	2.828	509	0.40	0.23	0.08	0.29
7	Take exams	0.661	0.044	−0.322	482	0.37	0.17	0.18	0.28
5	Do mathematics	0.346	0.046	1.552	468	0.22	0.21	0.21	0.35
14	Get assistance	0.064	0.050	−0.497	449	0.13	0.19	0.24	0.44
10	Participate in the classroom	−0.031	0.044	0.868	502	0.17	0.09	0.11	0.63
3	Speak	−0.048	0.044	−0.892	508	0.17	0.07	0.11	0.65
8	Participate in sport activities	−0.302	0.053	0.573	421	0.11	0.08	0.07	0.74
9	Participate in practical subjects	−0.334	0.053	0.343	417	0.11	0.04	0.06	0.80
12	Participate in practical activities during breaks	−0.496	0.055	−1.000	469	0.08	0.05	0.03	0.84
16	Interact with staff	−0.578	0.055	−1.754	507	0.06	0.08	0.06	0.80
11	Participate in social activities during breaks	−0.869	0.080	−1.580	506	0.03	0.00	0.02	0.95
15	Access the school	−1.002	0.089	−1.363	490	0.02	0.01	0.02	0.95
13	Go on field trips	−1.049	0.079	−1.431	368	0.02	0.07	0.05	0.86

“Remember things” the most difficult item and “Go on field trips” the easiest item

The SSI four-step rating scale:

a) 1: Unfit when the student perceives that the school environment needs to be modified but has not received any adjustments

b) 2: Partial fit when the student perceives that the school environment needs to be modified although some adjustments have already been received

c) 3: Good fit when the student has received needed adjustments and is satisfied with them

d) 4: Perfect fit when the student perceives that the school environment fit is ideal and the student does not need any adjustments at all

### Dif

**Fig. 3:**
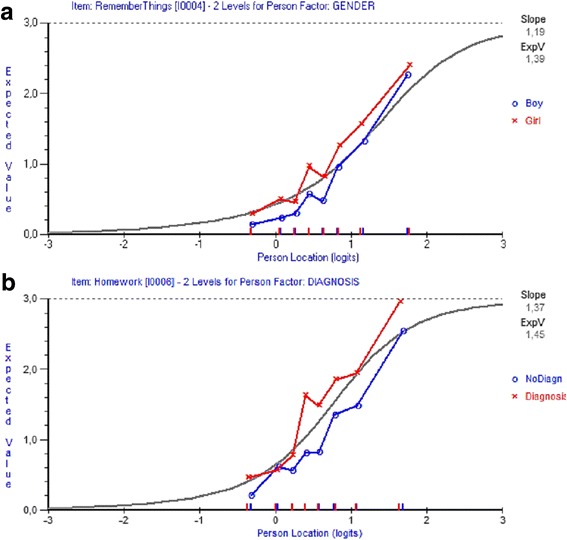
Graphical comparison between observed values of boys and girls in item Remember things (**a**) and between students with and without a medical diagnosis in item Homework (**b**) in eight class intervals, displaying DIF (Fig. 3)

Item 4 “Remember things” showed uniform DIF for gender with a significant *p*-value after Bonferroni adjustment (F-ratio 11.04, *p* < 0.001) (Fig. 3). The responses of girls were consistently higher along the trait than those for boys, indicating a higher score of perceived student-environment fit despite the same location on the latent construct. Item 6 “Do homework” showed uniform DIF with tendency to non-uniform DIF, for diagnosis with a significant *p*-value after Bonferroni adjustment (F-ratio 18.01, *p* < 0.000) (Fig. 3). The responses of students with a medical diagnosis were higher along the trait than those for students without diagnosis, indicating a higher score of perceived student-environment fit, despite the same location on the latent construct.

Splitting item 4 “Remember things” for gender and item 6 “Do homework” for diagnosis did not result in any notable changes regarding overall fit to the model (*chi sq.* = 137.72; *df* = 126; *p* = 0.22, item-fit: mean = −0.16, SD = 1.24 and person-fit: mean = −0.25, SD = 0.73) and no additional DIF was demonstrated. Thus, the original data set was retained in further analyses. [Fig. 3].

Graphical comparison between observed values of boys and girls in item Remember things (A) and between students with and without a medical diagnosis in item Homework (B) in eight class intervals, displaying DIF.

### Response category functioning

**Fig. 4:**
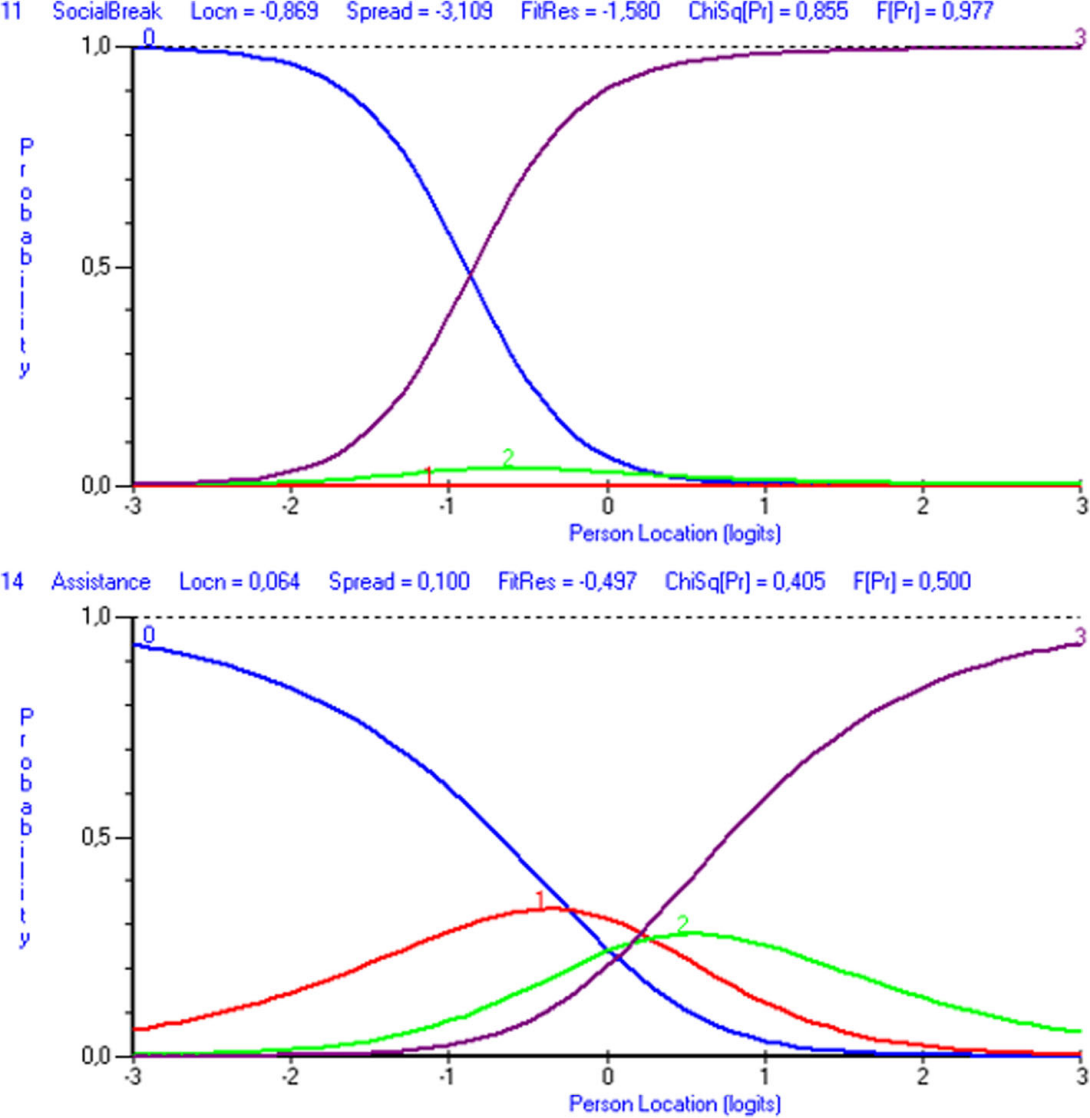
Response category functioning of SSI items

All items had disordered thresholds indicating issues with the categorization of the SSI items. The estimates of thresholds did not form distinctive regions of the continuum. Item 11 “Participate in social activities during breaks” had the most disordered thresholds and item 14 “Get assistance” had the best functioning ones. The probability of obtaining a score of 2 (partly fit) and 3 (good fit) was never most likely for students in item 11 (Fig. 4). Item 14, demonstrated a better functioning and only one category was disordered. When investigating the category response proportion, it was obvious that the highest proportion of students was rated as unfit (score 1) or perfect fit (score 4) in all items, see Table 2.

Item 11 “Participate in social activities during breaks” (upper picture) displaying disordered thresholds between categories 0-1 (score 1-2), 1-2 (score 2-3) and 2-3 (score 3-4) and item 14 “Get assistance” (lower picture) displaying disordered thresholds between categories 1-2 (score 2-3) and 2-3 (score 3-4). Category probability curves show the probability of observing each category relative to the location on the measured continuum of student-environment fit (x-axis).

### Unidimensionality and locally independence

The residual correlation matrix revealed that all correlations between items were under 0.3, demonstrating locally independence among the SSI items, i.e. no response dependence. The first principal component identified by the PCA had an eigenvalue of 1.69, explaining 10.55% of the variance. No additional structures were present since no associations were found in the data after the “Rasch factor” was extracted when investigating the PCA with the varimax rotation. Taken together, the overall fit and the PCA results support a unidimensional underlying construct.

## Discussion

This study was conducted to examine the measurement properties of the SSI among students with special educational needs in regular high school, a previously unevaluated field of use of the SSI. Data were found, in general, to be in accordance with the Rasch model, providing support for the internal construct validity of the SSI. However, areas for potential improvement were also identified where the SSI rating categories with the disordered thresholds are of most concern.

The findings indicated that the SSI captures three different levels of student-environment fit. The minimum requirement of this kind of assessment is to separate people into two groups (high and low level of attribute) [32]. Since the SSI is able to separate about three levels of performance, the sample may be classified into meaningful categories of high, medium and low experienced student-environment fit. The SSI three-step rating scale in earlier versions also separated students into three levels of student-environment fit and the new, four-step, rating scale was proposed to increase the separation [17]. This was not confirmed in the present study, perhaps because this new group of students had a different type of difficulties than previously investigated. Positively, the sample presented a good, overall, person-fit and the overall item-fit was acceptable. In clinical practice, it is important that the assessment is appropriately targeted to the population being assessed for adequate measurement [22, 25, 26] and satisfyingly, the targeting of the SSI items was good for this new group of students. Item 4 was the most difficult item for the sample, and with respect to the existing difficulties among the students, this was in accordance with the hypothesis. However, some more difficult items would probably increase the psychometric properties of the assessment [23] since the present sample exhibited a higher mean location than included items. Item 2, “Read”, showed signs of misfit, though these were non-significant. This observation may not convert into other populations, but if so, the misfit should be thoroughly investigated since reading is a highly important activity in school, and thus an essential item in the SSI.

Support for unidimensionality and local independence was satisfying since both multidimensionality and response dependency are serious threats of the psychometric properties of an assessment and implies that responses to an item depends on responses to other items or that the scale reflects more than one latent trait [26, 31].

The SSI rating categories did not work as intended for this group of students since all items had, more or less, disordered thresholds. Investigation of the response distribution confirmed that category 1 (score of 2 - partial fit, student has adjustment but additional adjustments are needed) and 2 (score of 3 - good fit, student has necessary adjustments) were rarely used. Both categories imply that adjustments have already been made in the environment to increase the student-environment fit. Thus, this indicates that students had not received needed adjustments, which was also demonstrated earlier for this group of students in Swedish schools [33] as well as internationally [19]. Even though laws and legislation [9, 34] entitle all students to participate in and acquire education, students with special educational needs do not seem to sufficiently obtain individualized support which would enhance and improve their performance and participation in school activities as a result of improved student-environment fit. Item 14 “Get assistance”, had the best category functioning and the most equal distribution of scores among the different categories. This indicates that if all assigned categories are not used, in this study due to absence of environmental adjustments, it might be a reason for disordered thresholds [35, 36]. Despite issues with the categorization of the scale, it is essential for the clinical utility of the SSI to keep all four categories since it is used both as an assessment of level of student-environment fit and as an evaluation of implemented adjustments [15]. Category 1 (score of 2) and 2 (score of 3) are of great value to see whether implemented adjustments fulfilled their purpose or whether the students are in need of further adjustments. The present study was based on data from an initial SSI assessment and further investigation of the category functioning should include data from SSI evaluation of implemented adjustments. Thus, further studies are necessary to investigate the underlying cause of why two of the four categories are not used as intended. If the reason for non-use of category 1 (score of 2) and 2 (score of 3) is not due to absent adjustments, the scale needs to be revised.

Item 4 “Remember things” showed uniform DIF regarding gender and item 6 “Homework” showed DIF among students with and without diagnosis. When DIF is present the observed group differences, at least partially, reflect something other than the latent trait [21, 37, 38] and comparison between boys and girls or between student with and without diagnosis is not completely reliable in the specific items. ANOVA statistics and the ICC curve were used in the present study since DIF analyses’ actual power is affected by sample size, the distribution of persons in relation to the location of items and the distribution of residuals [38]. Additionally, an item split was performed in order to investigate the DIF further [23, 38]. DIF does not necessarily imply clinical significance, and theoretical and practical issues should be considered before adjustments are made [39]. Removal of item 4 was not considered an option since much school activity, as well as everyday life, involves executive functions such as planning and remembering. The same applies for item 6 as homework is an integral part of schooling. Since the item split confirmed that the DIF did not affect the overall psychometric properties, the decision to keep the original SSI items was supported. Item Remember things might have different meanings for girls and boys, which might be reflected in the scoring of the item. Girls are generally more committed in their schoolwork [39] and spend more time doing school-related activities than boys [40]. This may indicate that they more often have self-initiated strategies for planning and remembering details related to school activities, which might have caused the detected DIF. What caused the DIF in item Homework might be related to that having a medical diagnosis is associated with increased odds of receiving support in Swedish schools [41]. The support may include specific assistance regarding the whole school activity of Homework, such as planning and modifications of tasks or instructions. This assistance might have influenced the higher scoring among students with a diagnosis, even though the students had the same level of student-environment fit as those without a diagnosis. The detected DIF should be investigated further to evaluate its clinical effect and whether they are also present in other samples.

### Methodological considerations

The Rasch analysis was chosen since it facilitates disclosure of measurement issues that may not be easily detected by traditional analyses [26]. Another advantage is that students with missing responses in some items could be included since the model does not require complete data to estimate person parameters [21, 22]. The skewness in used SSI rating categories was a limitation which might have negatively affected the evaluation of the scale. A sample generating data with more equal distribution among used rating categories would be desirable in further studies.

## Conclusion

The findings provide support of construct validity of the SSI for use among regular high school students with special educational needs in order to provide and evaluate environmental adjustments. Assessing student-environment fit to be able to provide environmental adjustments for students is essential, and for this evaluation the SSI could be used as a valuable tool by personnel at schools and student health units. However, the items with the detected DIF and the SSI rating scale with its disordered thresholds needs to be further scrutinized.

## Abbreviations

ADHD: Attention deficit hyperactivity disorder
ANOVA: Analysis of variance
DIF: Differential item functioning
ICC: Item characteristic curve
PCA: Principal component analysis
PSI: Person separation index
SD: Standard deviation
SSI: School setting interview
